# Multiphasic Reaction Modeling for Polypropylene Production in a Pilot-Scale Catalytic Reactor

**DOI:** 10.3390/polym8060220

**Published:** 2016-06-14

**Authors:** Mohammad Jakir Hossain Khan, Mohd Azlan Hussain, Iqbal Mohammed Mujtaba

**Affiliations:** 1 Department of Chemical Engineering, Faculty of Engineering, University of Malaya, Kuala Lumpur 50603, Malaysia; jakirkhanbd@gmail.com; 2 UM Power Energy Dedicated Advanced Centre (UMPEDAC), Wisma Research & Development, University of Malaya, Kuala Lumpur 59990, Malaysia; 3 Chemical Engineering Division, School of Engineering, University of Bradford, Bradford BD7 1DP, UK; I.M.Mujtaba@bradford.ac.uk

**Keywords:** polypropylene production, multiphasic model, CFD, pilot scale experimental validation

## Abstract

In this study, a novel multiphasic model for the calculation of the polypropylene production in a complicated hydrodynamic and the physiochemical environments has been formulated, confirmed and validated. This is a first research attempt that describes the development of the dual-phasic phenomena, the impact of the optimal process conditions on the production rate of polypropylene and the fluidized bed dynamic details which could be concurrently obtained after solving the model coupled with the CFD (computational fluid dynamics) model, the basic mathematical model and the moment equations. Furthermore, we have established the quantitative relationship between the operational condition and the dynamic gas–solid behavior in actual reaction environments. Our results state that the proposed model could be applied for generalizing the production rate of the polymer from a chemical procedure to pilot-scale chemical reaction engineering. However, it was assumed that the solids present in the bubble phase and the reactant gas present in the emulsion phase improved the multiphasic model, thus taking into account that the polymerization took place mutually in the emulsion besides the bubble phase. It was observed that with respect to the experimental extent of the superficial gas velocity and the Ziegler-Natta feed rate, the ratio of the polymer produced as compared to the overall rate of production was approximately in the range of 9%–11%. This is a significant amount and it should not be ignored. We also carried out the simulation studies for comparing the data of the CFD-dependent dual-phasic model, the emulsion phase model, the dynamic bubble model and the experimental results. It was noted that the improved dual-phasic model and the CFD model were able to predict more constricted and safer windows at similar conditions as compared to the experimental results. Our work is unique, as the integrated developed model is able to offer clearer ideas related to the dynamic bed parameters for the separate phases and is also capable of computing the chemical reaction rate for every phase in the reaction. Our improved mutiphasic model revealed similar dynamic behaviour as the conventional model in the initial stages of the polymerization reaction; however, it diverged as time progressed.

## 1. Introduction

The operational performance of the Fluidized Bed Reactors (FBRs) depends on their capacity to execute many multiphasic chemical reactions, uniform fluid mixing, a higher rate of heat and mass transfers, and operating in a continuous state [1,2,3,4]. Consequently, a lot of interest has been generated by the propylene polymerization model in an FBR [5,6,7]. In the industrialized gas-phase polypropylene FBR, smaller particles of the Ziegler-Natta catalyst along with triethyl aluminium are continuously charged in the bed reactor and they react with the various reactants for producing a wide distribution of the polymer particles. Several studies have indicated that the foremost aim of engineering the gas-phasic olefin polymerization reaction is to comprehend the way the reaction mechanism works, along with studying the physical transportation process, the reactor configurations and the reactor operational conditions, which can influence the properties of the polymer product [8,9,10]. It should be noted that the polymer products in the FBRs exhibit several types of properties, such as the morphological property and the molecular property [10,11,12,13,14]. Generally, the polymerization processes are classified as homogeneous and heterogeneous processes. In the homogeneous polymerization process, the reaction takes place in a single phase, while the polymerization takes place in different phases in a heterogeneous process. Hence, the heat transfer, the inter-phasic mass transfer, and the chemical reaction are very important to study [15,16,17,18,19,20]. Moreover, the multiphasic properties are connected to the industrial-scale polymerization reactor behavior from the pilot scales and are greatly impacted by the operational conditions of the reactor, such as gas–solid flow fields (*viz.*, the gas and the solid fractions). Due to this, detailed modeling describing the pilot-scale phenomenon is a very difficult task. The modeling of the pilot-scale FBR should take into consideration the complicated two-phase gas–solid flow, the interaction between the particles and the particle-reactor, along with microscale phenomena such as the chemical interactions and the kinetic reactions between catalyst-active sites and the molecular movement and particle collision. A multiphase reaction approach serves to solve the problems described above and establish the relation between the multiphasic polymerization rate and the operating conditions.

There have been very few research articles describing the pilot-scale, multiphase olefin chemical polymerization process. In the heterogeneous systems, the polymerization reaction takes place during the occurrence of the various phases that have an inter-phase mass, heat transfer and the chemical reactions. The actual modeling approach should incorporate the complicated gas-solid flow characteristics, kinetics of the heterogeneous polymerization reaction and different heat and mass transfer procedures. There are several protocols that describe the hydrodynamics of the polyolefin FBR. Some researchers [21,22,23] took into account the polyolefin FBR along with the well-mixed reactor. The authors compared their model and the uniformly mixed model under steady-state parameters and observed that the even-mixed model did not present any substantial error while predicting the monomer amount in the fluidized bed reactor and the temperature in comparison to the developed mathematical model. In their study [24], Alizadeh *et al.* (2004) described a gas–solid model wherein the reactor consisted of the emulsion and the bubble phase. They hypothesized that the polymerization took place in the emulsion phase only as the bubble phase was free of solids. A heterogeneous three-phase model was proposed by Caliani *et al.* (2006) [25], in which they considered the emulsion, bubble, and particulate phase having the plug flow behavior. In their work, Hatzantonis *et al.* (2000) [26] presumed that a reactor which is comprised of the mixed bubble and emulsion phases can be divided into many well-mixed, solid-free sections in a series. Generally, the polymers and the gaseous phases present in the FBR are considered to be evenly mixed. However, in several huge industrial FBRs, particle separation is seen to occur, indicating that particle dispersal varies with relation to the height of the bed. Also, it is noted that particle segregation could appear in the FBRs, which are run at very low gas-flow velocities (*viz.*, *u*_g_ ≈ 0.2 m/s), when the reactor contains larger particle sizes or greatly differing particle densities. A tank-in-series model was proposed by Satish *et al.* (2005) [27] to depict the reactor hydrodynamics. Harshe *et al.* (2004) [28] developed a thorough mathematical approach which was based on a mixing cell for simulating the transient behavior of the polypropylene FBRs. This model used the population balancing steady-state equations, along with incorporating the complex multisite, multi-monomer, polymerization kinetics. Also, Ibrehem *et al.* (2008) [11] suggested that the bed could be comprised of the bubbles, emulsion, cloud, and solid phases and also took into account the polymerization reactions which occurred in the emulsion phase and the solid phase. This model considered the influence of the type of catalyst particle and particle porosity on the reaction rate.

In all of the above-mentioned models, the authors presumed that no chemical reaction occurred in the gas bubble phase. However, Kiashemshaki *et al.* (2006) [22] presented a study, where they had sectioned the reactor in four serial sections, where every section contained the bubble gas phase as the plug flow and the emulsion phase as the uniform-dispersed phase. The system was modeled at the steady-state condition and it was hypothesized that the polymerization reaction occurred in the bubble and the emulsion phases.

Dompazis *et al.* (2005) [29] described a complex multi-scale and multi-compartmental dynamic model for analyzing the degree of solid dispersal in the catalytic olefin-polymerizing FBRs. This model used the “linking” model for four separate time and length scales, *i.e.*, the kinetics model, the single particle model and the multi-zonal mixing models. However, they were unable to couple the four models at their individual scales. Moreover, they implemented the integrated CFD–PBM–PMLM model for describing the gas–solid flow fields in the FBRs.

In our study, we aim to develop a novel polyolefin-based engineering process which minimizes the computational and the experimental attempts in the presence of a novel pilot-scale experimentation design. The study includes a modeling and a pilot-scale experimental validation, for designing a high-performance production system with additional advantages. As the multiphase model helps in the prediction of the relation between the PP (polypropylene) production rate and the reactor operational parameters, it is possible to develop some novel PP production processes that possess very good productivity and it is also possible to obtain their processing parameters in advance, which would help in their industrial and experimental development.

Moreover, in our study we have also employed the homopolymerization CFD scheme for understanding propylene homopolymerization in comparison to the heterogeneous Ziegler-Natta catalyst in the FBRs. We have assumed that the heat and mass transfer resistance between the emulsion gas and the polymer particles are almost negligible. Hence, we have carried out a comprehensive and extensive study for the gas–solid phase conversion and bubble formation caused by the hydrodynamic behavior, and an improved multiphase model was proposed to examine the effect of major parameters on the presumed bed reactor process variables and the polymer properties.

## 2. The Reactions and Kinetic Model for Polymerization

In our study, we have considered a complex catalytic (Ziegler-Natta catalyst) reaction mechanism for describing the propylene homopolymerization kinetics. The polypropylene production rate factors were explained using the momentum method. The necessary mass balance equations in the case of the reacted monomers (that are described by a sequence of differential and algebraic equations) were applied separately for the different emulsion and the bubble phase, as the plug flow reactor contains the very active sites of the catalyst. This was a better depiction of the situations faced by the heterogeneous Ziegler-Natta catalysts.

The Euler-Euler technique has been introduced for the analysis of the interphase phenomena taking place in the fluidized conditions. In this technique, the phases are mathematically modeled as the interpenetrating continua. As the phase volume is not taken over by other phases, this technique uses the theory of the phase volume fraction. The phasic volume fractions are supposed to be a continued function of space and time, with their summation equal to 1. The conservation equations, in the case of every phase, are derived for obtaining the equations, and they have analogous structures for the phases. The equations can be terminated after constitutive relations have been provided, and these are derived from the empirical statistics or by using the kinetic theory based on granular flow [30,31]. In the ANSYS FLUENT, two different multiphasic models can be obtained, from which the Volume of Fluid (VOF) model and the Eulerian model are used and integrated to form the mathematical models [32,33,34,35,36]. One of the most complicated multiphasic models in the ANSYS FLUENT is the Eulerian model. This model contains a group of momentum and continuity equations for every phase. The coupling can be possible by the pressure and the interphase exchange coefficients. The way the model handles the coupling is based on the categories of phases that are involved, *i.e.*, the granular (gas–solid) flows are treated differently as compared to the non-granular (fluid–fluid) flow. This study obtains the properties by applying the kinetic theory for examining the granular flow. The mixture which is being modeled also affects the exchange of momentum between the phases. Moreover, we have also used the ANSYS FLUENT’s feature of User-Defined Functions (UDF) that permits the customization of the momentum exchange calculation.

Though the polymerization mechanism is similar in both phases, the reaction rate between the bubble and the emulsion phase are very different. This is mainly because the dynamic two-phase model consists of varying concentrations of the solids in every phase and also differs in the amount of polymer present in the bubble (Vpb) and emulsion phases, which has been elaborated on in Section 4.2. Variations in the catalyst flow rates in the emulsion and the bubble phases result in differing reaction parameters for both the phases and influence the temperature, and production rate, along with the monomer conversion in these two phases. Applying the Eulerian multiphasic model along with the kinetic model helps in the analysis of the fluidized beds as certain mathematical hypotheses are important for developing the comprehensible reaction models, which are explained in further detail in the subsequent sections.

In our study, we have presumed that the main consumption of the monomer is only in the polymerization reaction and hydrogen consumption is through the transfer of hydrogen to the reaction. Hence, the consumption rate for the components (in the case of the monomer and the hydrogen) is obtained as follows:

The generalized equation describing the rate of the *r*_th_ catalytic reaction is as follows:

(1) $$R_{r}=k_{f,r}\left(\prod_{i=1}^{N_{g}}{\left[G_{i}\right]}_{ct}^{{\eta^{\prime }}_{i,g,r}{}^{\prime }}\right)\left(\prod_{j=1}^{N_{s}}{\left[S_{J}\right]}_{ct}^{{\eta^{\prime }}_{j,s,r}}\right)$$

For the forward rate coefficient for reaction (*r*_th_), the $k_{f,r}$ is computed by using the Arrhenius expression:

(2) $$k_{f,r}=A_{r}T^{\beta_{r}}e^{-E_{r}/RT}$$

where

*A*_r_ = pre-exponential factor (consistent units) $\beta_{r}=$ temperature exponent (dimensionless)
*E*_r_ = activation energy for the reaction (J/kmol)
*R* = universal gas constant

It is logical to use the specific method to characterize the rate expression in pressure-dependent reactions [37,38]. The gas-phase polymerization reaction is one in which the temperature and pressure are such that the reaction takes place between Arrhenius maximum-pressure and minimum-pressure limits, and as a consequence is no longer exclusively dependent on temperature.

However, based on the above equation, the net molar rate for the consumption or the production of specific species in various phases can be described as:

(3) $$R_{i,j(bu)}=\sum_{r=1}^{N_{bu}}B_{i,r}^{\prime \prime }\;B_{i,r\;r}^{\prime }\;\;i=1,2,3\ldots ..N_{\mathrm{bu}}$$

(4) $$R_{i,j(eu)}=\sum_{r=1}^{N_{eu}}E_{i,r}^{\prime \prime }\;E_{i,r\;r}^{\prime }\;\;i=1,2,3\ldots ..N_{\mathrm{eu}}$$

(5) $$R_{i,j(ct)}=\sum_{r=1}^{N_{ct}}C_{i,r}^{\prime \prime }\;C_{i,r\;r}^{\prime }\;\;i=1,2,3\ldots ..N_{\mathrm{ct}}$$

For monomer:

(6) $$R_{i,p}=\sum_{j=1}^{N_{as}}{(M_{i})Y(0,j)k_{p}\left(j\right)}_{}\;i=1$$

For hydrogen:

(7) $$R_{i,h}=\sum_{j=1}^{N_{as}}{(M_{i})Y(0,j)k_{h}\left(j\right)}_{}\;i=2$$

The reaction rate coefficients were taken from the literature and are given in Table 1 [11,15].

In our study, we considered the impact of temperature (*i.e.*, the activation energy) on the polymerization kinetics for the polymerization reactions only. There have been many reports which have stated that in the cases where the Ziegler-Natta particles are very small and their activity is not very high (low or moderate rate of polymerization), then the mass and the heat transfer resistance present in the polypropylene and within the unreacted solid and the gas particles play an insignificant role and they will not influence the reactor behavior or even the polyolefin properties [39].

The FBRs are not very ideal and are tough to characterize due to the presence of complicated mixing and the contact flow patterns, the transportation phenomenon and the various polymerization reactions. Several researchers have tried to model this type of non-ideal system by developing numerous mixing models for describing this kind of behavior. These types of reactors, generally, need to combine the hydrodynamics, kinetics, and transport phenomena for their modeling. In one study, the dynamic performance of the FBR was described by Choi and Ray (1985) [40], wherein they suggested a steady bubble-sized model which comprised the well-mixed emulsion phase along with a plug flow bubble phase. Researchers also developed a very simple evenly mixed model by hypothesizing that the reaction contained an unobstructed transfer of heat and mass within the emulsion and the bubble phases [21]. In this study, we have adopted the unified modeling method for studying the gas–solid fluidization. A bubble-emulsion phase flow model has been developed for describing the dynamic behavior, which involves the multidimensional flow pattern and the multifaceted mixing of the polymer, PP, and gaseous phase FBR. For estimating the mean value of the bed voidage and the energy and mass balance equations, we have derived the dual-phasic model by combining the previously described kinetic developments and the dynamic two-phase model.

### 2.1. The Multiphasic Hydrodynamic Models

In this model, it has been assumed that the bubble phase does not contain any solids and the emulsion phase continues at minimal fluidization conditions. However, the emulsion phase voidage may differ from that in the minimum fluidization conditions. Additionally, the bubble phase could also contain different solid particle fractions [41]. Using this idea as the basic step, Cui *et al.* [41] suggested the dynamic inter-phase flow for studying the hydrodynamics of the fluidized bed (the concentrations of the solid particles vary in the emulsion and the bubble phases depending on the gas velocity). Hypothesizing the emulsion phase minimal fluidization conditions in the PP reactor (for a conventional two-phase model) is unrealistic, hence, in this study, the dynamic two-phase flow of the fluidized beds, as suggested by Shamiri *et al.* (2010) [15], has been incorporated along with the CFD model. This would help improve the multiphasic model used in our study and would also help in the calculation of the mean bed voidages. The correlation required for the estimation of the bubble volume fraction in the fluidized beds, the emulsion and the bubble phase velocities, the emulsion phase and the bubble phase voidage, and the mass and heat transfer coefficients in the case of the two-phase model and the steady bubble-sized model have been summarized in Table 2.

### 2.2. The Emulsion Phase Model

In their study [44], Hassani *et al.* (2013) developed a simple well-mixed model in which they assumed that the bubbles are very small and possess an unobstructed heat and mass transfer within the emulsion, and the bubble phases, the composition and the temperature were homogenous in the gaseous phase present in the fluidized bed. A good solid mixing is vital for ensuring a consistent distribution of product quality and maintaining a constant solid temperature or concentration in the bed. Also, the hydrodynamic elements such as bed porosity and bubble motion directly affect the solid flow mixing/pattern in the bed. It is also suggested that developing programming codes based on the requirement to elucidate the unsteady dynamic helps reduce the CPU (central processing unit) time. In this study, we have also developed coding through the use of user-defined functions (UDF) to serve this purpose. Some significant assumptions also made for this modeling are mentioned below:

1. The heat and the mass transfer rates in the bubble and the emulsion phase were very high and the bubbles were very small; hence, the polymerization reaction is a single-phase reaction, while the reactor is believed to be a single-phasic (emulsion phase), well-mixed type of reactor.
2. The emulsion phase continues in minimum fluidization conditions.
3. The bed consists of uniform composition and temperature.

Considering the above-mentioned assumptions, the energy-balance and the dynamic material equations in the case of the monomer and hydrogen concentration are written depending on the above assumptions. The equation for estimating the mole balance can be calculated as follows:

$$(V_{R}\varepsilon_{\min})\frac{d\left[M_{i}\right]}{dt}=U_{0}A\left({\left[M_{i}\right]}_{in}-\left[M_{i}\right]\right)-R_{v}\varepsilon_{\min}\left[M_{i}\right]-\left(1-\varepsilon_{\min}\right)R_{p}$$

The energy-balance equation considers the monomer internal energy as negligible. Therefore, the primary conditions that help in solving the equations are described below:

(8) $$\begin{array}{l} \left[\sum_{i=1}^{m}\left[M_{i}\right]C_{\mathrm{pi}}V\varepsilon_{\min}+V\left(1-\varepsilon_{\min}\right)\rho_{\text{pp}}C_{p,\mathrm{sol}}\right]\frac{dT}{dt}=U_{o}A\sum_{i=1}^{m}\left[M_{i}\right]C_{\mathrm{pi}}\left(T_{\text{in}}-T_{r}\right)-U_{o}A\sum_{i=1}^{m}\left[M_{i}\right]C_{\mathrm{pi}}\left(T_{\text{in}}-T_{r}\right)\\ -R_{\text{vol}}\left[\sum_{i=1}^{m}\left[M_{i}\right]C_{\mathrm{pi}}\varepsilon_{\min}+\left(1-\varepsilon_{\min}\right)\rho_{\text{pp}}C_{p,\mathrm{sol}}\right]\left(T-T_{r}\right)+\left(1-\varepsilon_{\min}\right)\Delta H_{R}R_{p} \end{array}$$

### 2.3. The Bubble Phase Model

Shamiri *et al.* (2010) [15] proposed the constant bubble-sized model which assumes that the emulsion phase (or the dense phase) is present in the minimal fluidization conditions. This model was adapted in several earlier reports which studied the gas-phase olefin chemical polymerization reaction.

The hypotheses for the bubble phase model have been described below:

1. The fluidized bed contains two different phases, *i.e.*, the bubble and the emulsion phase, and the chemical reactions generally take place in the emulsion phase only.
2. The emulsion phase is believed to be mixed completely, at minimum fluidization, and it exchanges mass and heat at uniform rates with the bubble phase above the height of the bed.
3. The bubbles are spherically shaped and have varied sizes and are in a plug flow with a constant velocity.
4. The heat and mass transfer resistances which occur between the solid polymer and the gas in the emulsion phase are very small (*i.e.*, presence of very minute catalyst particles, low-to-moderate catalytic activity or very low polymerization rates).

Based on these hypotheses, the energy balance and the steady-state mass can be estimated to describe the difference in the temperature and monomer concentration present in the bubble phases. The equation for the mole balance in the case of hydrogen and the monomer is as described below:

(9) $$\frac{d{\left[M_{i}\right]}_{bu}}{dt}=\frac{K_{\vec{b}e}}{U_{bu}}({\left(\left[M_{i}\right]\right)}_{eu}-{\left[M_{i}\right]}_{bu})$$

Integration of the neighboring monomer concentration [M_i_]_b_ present in the bed helps in the estimation of the average concentration for the *i*^th^ monomer present in the bubble phases.

(10) [M¯i]=1H∫0H[Mi]budh=[Mi]eu+([Mi]±eu(in)[Mi]in)×UbuKb⇀eH(1±exp(-Kb⇀eHUbu)

The bubble phase energy balance is expressed by the following equation:

(11) $$\sum_{i=1}^{m}{\left[M_{i}\right]}_{bu}C_{pi}\frac{dT_{bu}}{dt}=\frac{H_{\vec{b}e}}{U_{b}}(T_{b}\_T_{c})$$

Integration of Equation (10) for the overall height of the bed estimates the mean temperature of the bubble phase, which can be expressed as:

(12) $${\bar{T}}_{bu}=\frac{1}{H}\int_{0}^{H}T_{b}dh=T_{eu}+\left(T_{in}-T_{eu}\right)\frac{U_{b}{\bar{C}}_{p}}{H_{\vec{b}e}H}\left(1-\exp(-\frac{H_{\vec{b}e}H}{U_{b}{\bar{C}}_{p}}\right)$$

where the mean heat capacity for the reacting participants is as follows.

(13) $${\bar{C}}_{p}=\sum_{N=1}^{Ni}{\left[{\bar{M}}_{i}\right]}_{bu}C_{pM_{i}}$$

The dynamic molar balance for the *i*-th component for the emulsion phase may derived as

(14) $$\begin{array}{l} (V_{eu}\varepsilon_{\min})\frac{d{\left[M_{i}\right]}_{eu}}{dt}=U_{eu}A_{eu}\varepsilon_{\min}\left({\left[M_{i}\right]}_{eu,in}-{\left[M_{i}\right]}_{eu}\right)\\ +\frac{V_{eu}\alpha_{eu}K_{\vec{b}e}}{\left(1-\alpha_{eu}\right)}\left({\left[M_{i}\right]}_{bu}-{\left[M_{i}\right]}_{eu}\right)-R_{v}\varepsilon_{\min}{\left[M_{i}\right]}_{eu}-\left(1-\varepsilon_{\min}\right)R_{i} \end{array}$$

The emulsion phase energy balance was expressed as

(15) $$\begin{array}{l} \left[\sum_{i=1}^{m}V_{\mathrm{eu}}\varepsilon_{\text{mn}}{\left[M_{i}\right]}_{eu}C_{\mathrm{pi}}+V_{\mathrm{eu}}\left(1-\varepsilon_{\min}\right)\rho_{\mathrm{pol}}C_{p,\mathrm{sol}}\right]\frac{dT_{eu}}{dt}=-\sum_{i-1}^{m}V_{\mathrm{eu}}\varepsilon_{\min}C_{\mathrm{pi}}\frac{d{\left[M_{i}\right]}_{eu}}{dt}\left(T_{e}-T_{r}\right)\\ +U_{\mathrm{eu}}A_{\mathrm{eu}}\varepsilon_{\min}\sum_{i=1}^{m}{\left[M_{i}\right]}_{eu,in}C_{\mathrm{pi}}\left(T_{e},in-T_{r}\right)-\frac{V_{\mathrm{eu}}\varepsilon_{\min}H_{\vec{b}e}}{\left(1-\varepsilon_{\min}\right)}\left(T_{e}-T_{r}\right)\\ +R_{v}\left((1-\varepsilon_{\min}\right)\rho_{\mathrm{pol}}C_{p,\mathrm{sol}}+\varepsilon_{\min}\sum_{i=1}^{m}{\left[M_{i}\right]}_{eu,in}C_{\mathrm{pi}})\left(T_{e}-T_{r}\right)+\left((1-\varepsilon_{\min}\right)\nabla H_{r}R_{p} \end{array}$$

The following equations have been used as initial conditions:

$${\left[M_{i}\right]}_{bu,z=0}={\left[M_{i}\right]}_{in}$$

$$T_{b}\left(z=0\right)T_{in}$$

$${\left[M_{i}\right]}_{eu,t=0}{\left[M_{i}\right]}_{in}$$

$$T_{e}\left(t=0\right)=T_{in}$$

### 2.4. The Inter-Phase Hydrodynamic Model

Generally, in the traditional constant bubble-sized and the well-mixed models, it is assumed that the emulsion would remain at its minimum fluidization ($\varepsilon_{eu}=\varepsilon_{\min}$) condition and the bubbles would be solid-free ($\varepsilon_{bu}=1$). However, these assumptions do not permit the prediction of the impact of the gas-solid dispersal on the actual reactions along with the mass/heat transfer rate which would be present in the beds at velocities which are greater than the minimal fluidization velocities. On the other hand, experimental and theoretical data have shown the presence of the solids in the bubble phase [45]. Also, (Abrahamson and Geldart, 1980) [45] stated that the emulsion phase would not stay at the minimal fluidization condition and it would also a greater gas concentration at greater velocities. When these two phases get mixed properly, it leads to an increased number of solid particles which enter the bubble phase and also more gas (propylene) that enters the emulsion phase, whereas it also leads to an increase in superficial gas velocities in the bed. The phase interface(s) can be tracked by applying the continuity equation to the volume fraction for one or more than one phase. The equation can be calculated for the *i*^th^ phase as follows:

(16) $$\frac{1}{{\left[M_{i}\right]}_{in}}\left[\frac{d}{dt}\right]\left(\alpha_{i}M_{i}\right)+\nabla .\left(\alpha_{i}M_{i}{\vec{v}}_{i}\right)=S_{\alpha_{i}}+\sum_{j=1}^{n}\left({\dot{m}}_{i.j}\pm {\dot{m}}_{j,i}\right)$$

where $\left({\dot{m}}_{\mathrm{i.j}}\right)$ refers to the mass transfer from the *i* phase to the *j* phase. By default, the source term on the right side of the equation would always be $S_{\alpha_{i}}$ = 0; however, it could also be stipulated by the constant value or by the user-defined mass source value for every phase particulate loading ($\left(\ell_{\text{pt}}\right)$), which also affects the phase interactions. Particulate loading can be defined as the emulsion phase’s mass density ratio to the mass density ratio for the bubble phase:

(17) $$\left(\ell_{\text{pt}}\right)=\frac{[M_{i}]\rho_{\mathrm{eu}}}{{\left[M_{i}\right]}_{\text{in}}\rho_{\text{pp}}}$$

The multiphasic model was studied for determining the behavior of the dynamic fluidized bed for the various important process parameters. This was conducted by using the software ANSYS 16.1 (ANSYS Inc., Berkeley, CA, USA), as this software provided a parallel and well-integrated computational service for estimating complicated multiphasic flows and the effect of the process parameters on the propylene production rate. We have applied the Eulerian-Eulerian method for simulating dynamic phase behavior. The built-in mathematical PBM (Population Balance Model) and the moment methodology were applied for evaluating the production rate of the polymer in actual reaction environments. To explain further, the second-order time method is applied for all transport equations, which include the mixture-phase momentum equations, all the species transport equations, the energy equations, the turbulence model, the phase volume fraction equation, the pressure-correction equation, and the granular flow model. It should be noted that solving a multiphasic system is quite complicated and it could encounter several stability and convergence issues. Instabilities generally arise from the poor initial field, and, hence, this requires a stable initial field. Moreover, the CPU time also poses a concern with respect to the transient issues; therefore, we considered the PC-SIMPLE option. In this study, the momentum equation which was used depends on the fraction volume of all the phases throughout the material characteristics. We have suggested the multiphasic mass transfer model which considers the mass transfer occurring between the species that belong to various other phases. In the model, rather than having a matrix type of data input, one needs to input the several mass transfer procedures. Every procedure then describes the mass transfer occurrence from a particular entity to other entities. An entity refers to either some species present in the phase or to the overall bulk phase if this phase contains no mixture in it. The mass transfer phenomena have been described using the user-defined functions, which have been developed. The dynamic multiphasic fluidized bed requirements have been explained using the following transport equations in Table 3.

In our present study, we have used the dynamic multiphase model, which was partially suggested by Khan *et al.* (2016) [43], and this model provided a better knowledge of the various hydrodynamic phenomena and also improved the quantitative knowledge of the real process. In any bubbling FBR, the upward movement of the bubbles can lead to better mixing of the solid particles within the emulsion or the dense phase. This can lead to a uniform concentration of the different particles and even temperature within the dense phase. Hence, a CFD-based pseudo-homogeneous model is also adopted for this phase. The gas bubbles move upwards in the bed with a fixed velocity while the particles move downwards, and they display an increase in size and mass when they flow in the downward direction. This justifies the use of the plug flow within the bubble phase. We have made the following assumptions for developing equations for the proposed improved model:

1. The polymerization reaction takes place in both the emulsion and the bubble phases.
2. The emulsion phase would be well mixed and it would not stay at minimal fluidization conditions.
3. We have assumed that the bubbles are spherically shaped and possess a uniform size. They have also been assumed to travel upwards in the fluidized bed in a plug flow with constant velocities.
4. The resistance of the mass and heat transfer within the gas and the solid particles present in the bubble and the emulsion phases have been assumed to be negligible (refers to very low or moderate catalytic activities [46]).
5. The agitation which results from the upwards flow of the bubbles leads to negligible radial concentrations and temperature gradient in the FBR.
6. Elutriation of the solids on the upper layer of the FBR is considered to be negligible.
7. It has been assumed that the size of the particles is constant within the bed.
8. The reactor uses materials that flow in a pseudo-homogeneous phase. The hydrodynamic features of the bed are defined using the average hydrodynamic properties of the existing phases (emulsion and bubble).

Considering the above-mentioned assumptions, we present the dynamic material balance equations for all components present in the FBR:

For bubbles:

(18) $$\begin{array}{l} {\left[M_{i}\right]}_{\mathrm{bu},(\mathrm{in})}U_{\mathrm{bu}}A_{\mathrm{bu}}-{\left[M_{i}\right]}_{\mathrm{bu}}U_{\mathrm{bu}}A_{\mathrm{bu}}-R_{v}\varepsilon_{\mathrm{bu}}{\left[M_{i}\right]}_{\mathrm{bu}}-K_{\text{be}}\left({\left[M_{i}\right]}_{\mathrm{bu}}-{\left[M_{i}\right]}_{\mathrm{eu}}\right)V_{\mathrm{bu}}\\ -\left(1-\varepsilon_{\mathrm{bu}}\right)\frac{A_{\mathrm{bu}}}{V_{\text{br}}}\int R_{i,\mathrm{bu}}dt=\frac{d}{dt}\left(V_{b}\varepsilon_{\mathrm{bu}}\right){\left[M_{i}\right]}_{\mathrm{bu}} \end{array}$$

For emulsion:

(19) $$\begin{array}{l} {\left[M_{i}\right]}_{\mathrm{eu},(\mathrm{in})}U_{\mathrm{eu}}A_{\mathrm{eu}}-{\left[M_{i}\right]}_{\mathrm{eu}}U_{\mathrm{eu}}A_{\mathrm{eu}}-R_{v}\varepsilon_{\mathrm{eu}}{\left[M_{i}\right]}_{\mathrm{eu}}-K_{\vec{b}e}\left({\left[M_{i}\right]}_{\mathrm{bu}}-{\left[M_{i}\right]}_{\mathrm{eu}}\right)V_{\mathrm{eu}}\\ -\left(\frac{1}{1-\varepsilon_{\mathrm{eu}}}\right)R_{i,\mathrm{eu}}=\frac{d}{dt}\left(V_{b}\varepsilon_{\mathrm{bu}}\right){\left[M_{i}\right]}_{\mathrm{eu}} \end{array}$$

Moreover, we have assumed that the mass transfer direction is from the bubble phase to the emulsion phase.

The energy balances can be expressed as for bubbles:

(20) $$\begin{array}{l} U_{\mathrm{bu}}A_{\mathrm{bu}}\left(T_{\mathrm{bu},(\mathrm{in})}-T_{r}\right)\sum_{i=1}^{m}{\left[M_{i}\right]}_{\mathrm{bu},(\mathrm{in})}C_{\mathrm{pi}}-U_{\mathrm{bu}}A_{\mathrm{bu}}\left(T_{\mathrm{bu}}-T_{r}\right)\sum_{i=1}^{m}{\left[M_{i}\right]}_{\mathrm{bu}}C_{\mathrm{pi}}-R_{v}\left(T_{\mathrm{bu}}-T_{r}\right)\\ (\sum_{i=1}^{m}\varepsilon_{\mathrm{bu}}C_{\mathrm{pi}}{\left[M_{i}\right]}_{\mathrm{bu}}+\left(1-\varepsilon_{\mathrm{bu}}\right)\rho_{\mathrm{pol}}C_{p,\mathrm{pol}})+H_{\vec{b}e}(T_{e}-T_{b})V_{b}-V_{b}\varepsilon_{b}\left(T_{\mathrm{bu}}-T_{r}\right)\sum_{i-1}^{m}C_{\mathrm{pi}}\frac{d}{dt}{\left[M_{i}\right]}_{\mathrm{bu}}\\ =\left(V_{b}\left(\varepsilon_{\mathrm{bu}}\sum_{i-1}^{m}C_{\mathrm{pi}}\frac{d}{dt}{\left[M_{i}\right]}_{\mathrm{bu}}+(1-\varepsilon_{\mathrm{bu}})\rho_{\mathrm{pol}}C_{p,\mathrm{pol}})\right)\right)\frac{d}{dt}\left(T_{\mathrm{bu}}-T_{r}\right) \end{array}$$

For emulsion:

(21) $$\begin{array}{l} UeuA_{\mathrm{eu}}\left(T_{\mathrm{eu},(\mathrm{in})}-T_{r}\right)\sum_{i=1}^{m}{\left[M_{i}\right]}_{\mathrm{eu},(\mathrm{in})}C_{\mathrm{pi}}-U_{\mathrm{eu}}A_{\mathrm{eu}}\left(T_{\mathrm{eu}}-T_{r}\right)\sum_{i=1}^{m}{\left[M_{i}\right]}_{\mathrm{bu}}C_{\mathrm{pi}}-R_{v}\left(T_{\mathrm{eu}}-T_{r}\right)\\ (\sum_{i=1}^{m}\varepsilon_{\mathrm{eu}}C_{\mathrm{pi}}{\left[M_{i}\right]}_{\mathrm{eu}}+(1-\varepsilon_{\mathrm{eu}})\rho_{\mathrm{pol}}C_{p,\mathrm{pol}})-(1-\varepsilon_{\mathrm{eu}})R_{p,\mathrm{eu}}\Delta H_{R}+H_{\text{be}}V_{\mathrm{eu}}(\frac{\alpha_{\mathrm{bu}}}{1-\alpha_{\mathrm{bu}}})(T_{e}-T_{b})-V_{b}\varepsilon_{b}\left(T_{eu}-T_{r}\right)\sum_{i-1}^{m}C_{pi}\frac{d}{dt}{\left[M_{i}\right]}_{eu}\\ =\left(V_{\mathrm{eu}}\left(\varepsilon_{\mathrm{eu}}\sum_{i-1}^{m}C_{\mathrm{pi}}\frac{d}{dt}{\left[M_{i}\right]}_{\mathrm{eu}}+(1-\varepsilon_{\mathrm{eu}})\rho_{\mathrm{pol}}C_{p,\mathrm{pol}})\right)\right)\frac{d}{dt}\left(T_{\mathrm{eu}}-T_{r}\right) \end{array}$$

### 2.5. Coupling Steps of MultiphasPhasic CFD-Based Reaction Model

**Figure 1:**
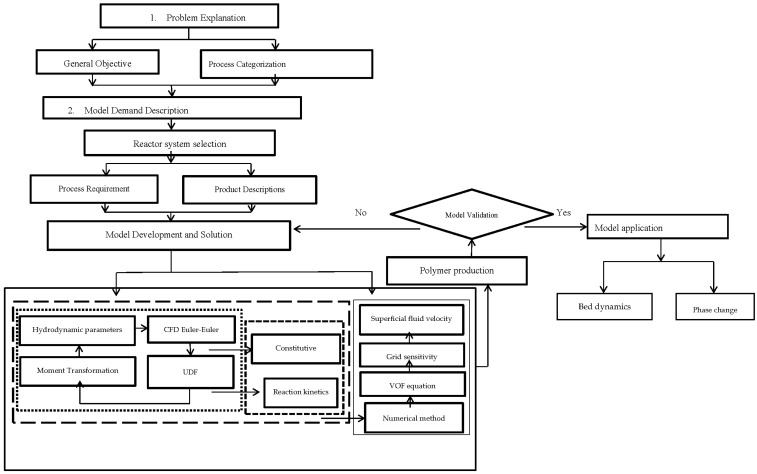
Steps of CFD-based multiphasic reaction model development.

To correlate the turbulence, population balance and energy equations in a multi-fluid UDF framework, a systematic CFD reaction kinetic coupled modeling framework application of multiphase polymerization in the fluidized bed reactor was executed. The CFD-based coupled model constitutes a flexible platform. Hence, its applications can be expanded to different polydisperse multiphasic FBRs by altering the geometry and constitutive equation. The generic model comprises four main steps, as shown in Figure 1:

- Problem definition
- Problem specification
- Model structure/solution
- Model applications

The concentrations of the species (propylene) for which the source term is a nonlinear function determine the stability of the UDF-coupled CFD simulation. This shows that the reaction rate is highly sensitive, and hence cannot be eliminated in the multiphasic reaction simulation procedure.

### 2.6. Grid Sensitivity Analysis

**Figure 2:**
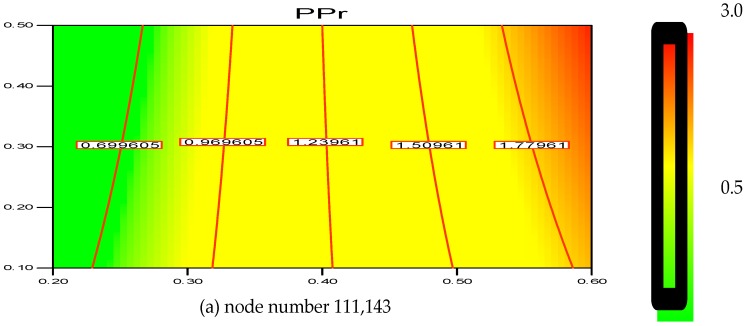

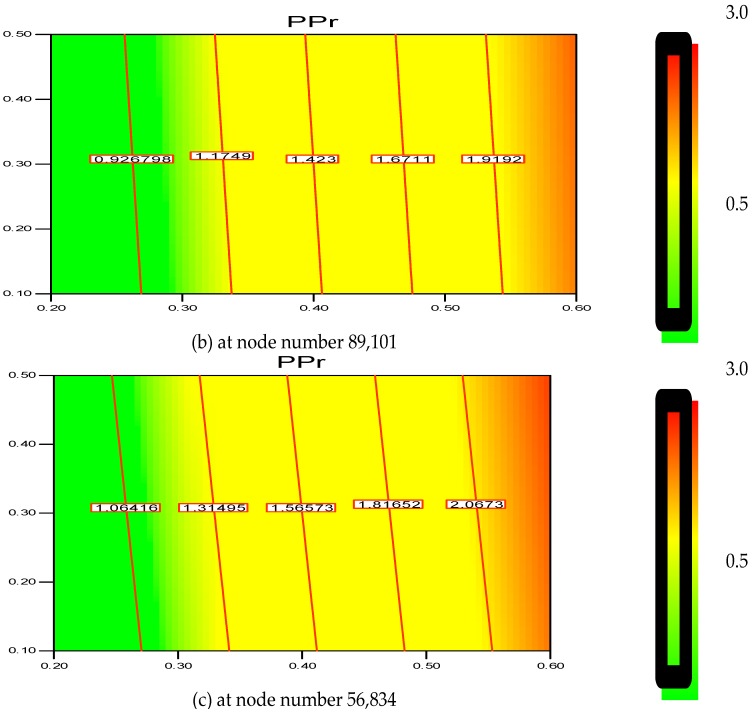
(**a**) Changes of the polymerization rate at node number 111,143 at various superficial gas velocities. Contour lines indicate the polymerization (%) changes; (**b**) Changes of the polymerization rate at node number 89,101 at various superficial gas velocities. Contour lines indicate the polymerization (%) changes; (**c**) Changes of the polymerization rate at node number 56,834, at various superficial gas velocities. Contour lines indicate the polymerization (%) changes.

The greater the resolution, the more independent the grid outcome is. This was confirmed with the help of a two-dimensional (2D) analysis that employs the boundary-and-gradient adaptation technique. In this procedure, the adjoined mesh points could be present in high-gradient areas in the inlet and fluidization regions. The response variations at three mesh resolutions with 56,834, 89,101 and 111,143 node numbers are shown in Figure 2a–c. The parameters considered for the simulation include 1.5 m of bed height, 1000 s real time and 0.2 m/s superficial gas velocity. Figure 1 demonstrates the three separate grids used to divide the 2D flow domain into square cells. Hence, it can be said that grid resolution plays an influential role for the response, as evident in Figure 2a–c.

**Figure 3:**
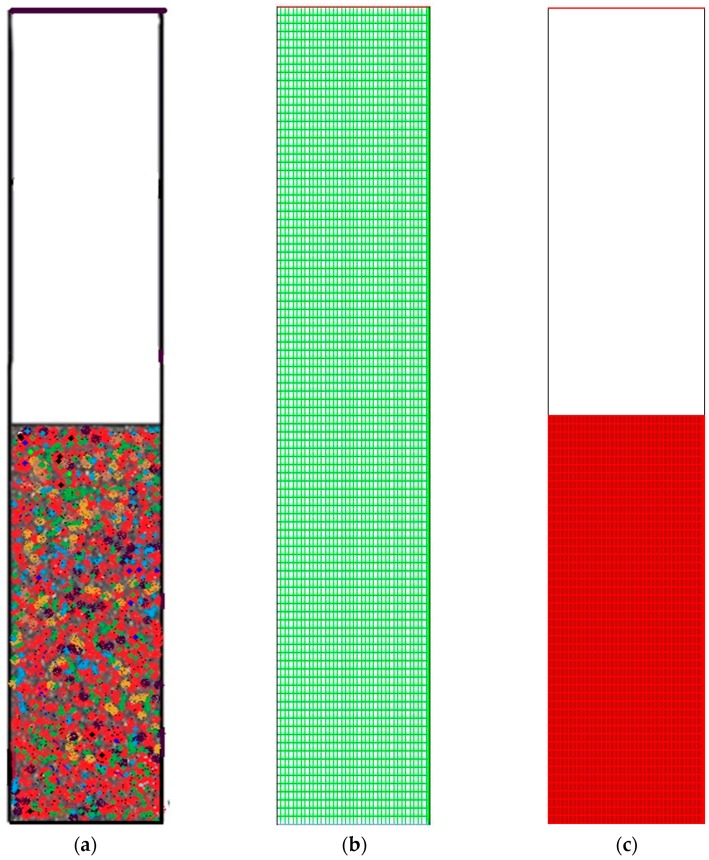
General computational domain and mesh generation. (**a**) Framework of the gas-phase fluidized bed polymerization reactor used in the simulation; (**b**) Generated mesh for the fluidized bed simulation; (**c**) Computational region marked.

Thus, according to the nodes’ variation, it is found that the polymerization percentage variation is in the range of 0.699%–1.779% when the node number is at 111,143. However, with the decrease in grid resolution (from node number 111,143 to 89,101), the response value also reaches a range of 0.926%–1.919%. Hence, the response calculation becomes less accurate as the node number decreases. Moreover, at node number 56,834 the polymerization percentage varies in wider range from 1.064%–2.067%. In this scenario, it has been verified that node number 111,143 should be considered as a compromised establishment for calculation and necessary accuracy. Thus, during the simulation on the pilot scale, sufficient grid convergence with a small polymerization difference from 0.699% to 1.779% at 111,143 nodes is required to achieve a more precise outcome. Figure 3 depicts the overall computational domain and mesh generation. Figure 3a shows a sketch of the fluidized bed packed with granulated particles. The meshing and the marked domain are shown in Figure 3b,c, respectively.

## 3. Experimental Facilities

**Figure 4:**
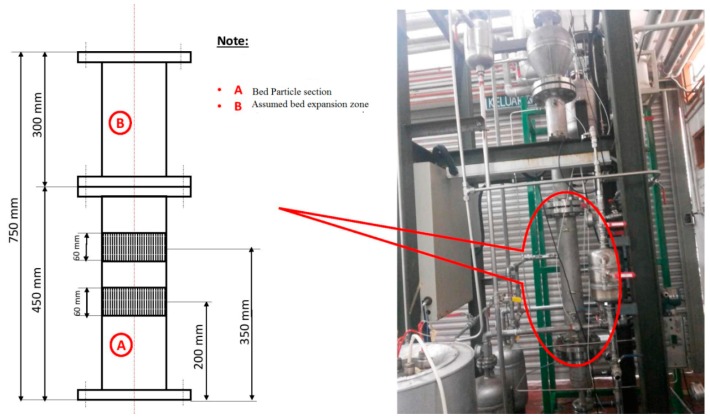
Image of the pilot-scale FBCR for polypropylene production where the experiments were conducted for this study (detailed dimensions have been shown in mm).

**Figure 5:**
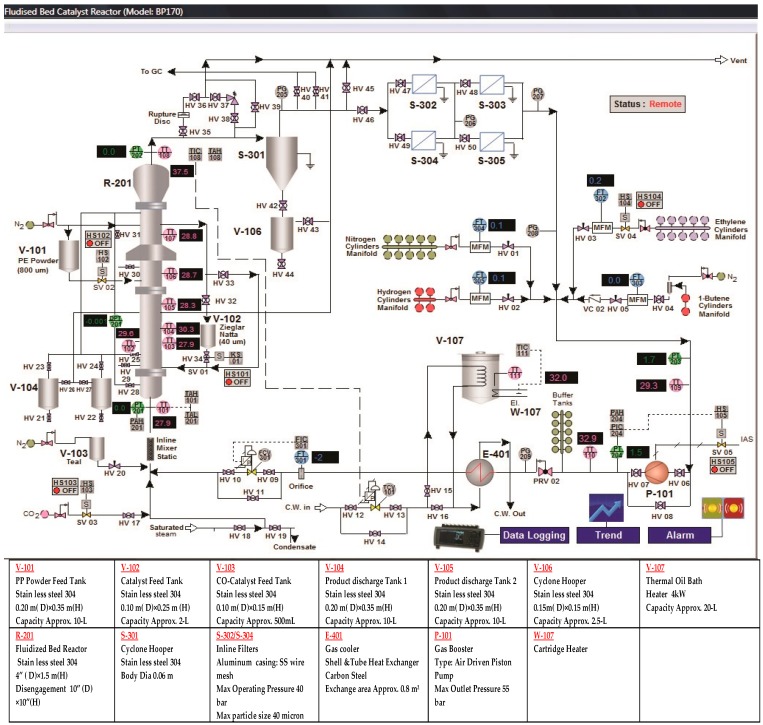
A Real-time data acquisition system for the pilot-scale FBCR for polypropylene production.

**Figure 6:**
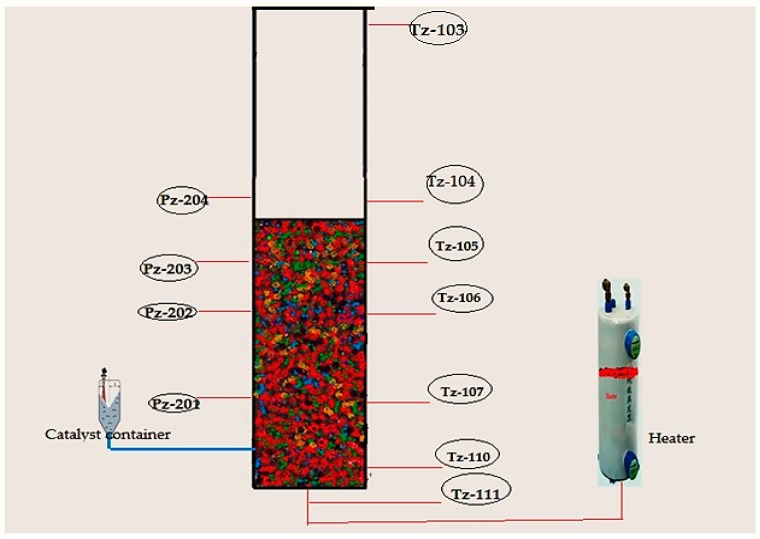
Pressure and temperature profile measurement scheme for real-time data acquisition system (fluidized bed has been shown before gas mixer introduction in the system).

A pilot-level fluidized bed reactor has been built in the pilot-scale Research Laboratory at the University of Malaya. The major aim of constructing this kind of experimental unit was to examine the catalytic polymerization reaction of the olefins at actual operating conditions which are similar to industrial parameters. In Figure 4, Figure 5 and Figure 6, we have described the picture, the data acquisition method and a detailed diagram of the pilot-level fluidized bed reactor.

This reactor consists of the fluidized bed and the product discharge zone. The reactor has an inner diameter of 10 cm while the fluidized bed zone height is 150 cm. Both the diameter and the height of the discharge zone are 25 cm. The catalyst particles have been introduced into the fluidized bed in the form of an injection at 9 cm above the gas distribution point. The product specimens were then withdrawn from three separate locations, *i.e.*, at the points which were 16, 26, and 40 cm above the position of the distributor plate. The polymer that is produced is discharged in a semi-continuous manner by opening the valve that is attached to the vessel at the point which is 5 cm over the gas distribution point. The gas distributor consists of a stainless steel plate which is perforated and consists of a fine mesh. The gas flow is controlled with the help of the control valve and is measured using the flow meter situated in front of the reactor. The fluidized bed reactor has one important requirement, wherein the recycled gas stream velocity should be enough so that the bed is always in a fluidized state.

Very pure quality raw material is needed for the catalytic olefin polymerization reaction to prevent the catalyst from being poisoned. The nitrogen, hydrogen and propylene have been purified in different purification systems (*i.e.*, Entegris Gate Keeper gas purifiers) for removing any traces of impurities of water vapor, oxygen, or carbon monoxide. For measuring the flow of hydrogen, nitrogen and propylene, three separate mass flow meters (Brooks, Hatfield, PA, USA) have been applied in the fresh feed streams.

For temperature measurements, it is hard to combine a high enough sample frequency to obtain a dynamic signal with the robustness of the equipment needed for industrial measurements. To overcome this problem we have fabricated seven temperature sensors at various points of the reactor. Secure and resilient pressure sensors with a high frequency of response have been set up at four positions (see in Figure 6). If a probe of appropriate size is selected, direct contact between the fluidized particles and the sensor can be prevented without interrupting the temperature and pressure signal. Also, the interaction between the highly reactive gaseous chemicals and the probe can be averted by directing a small purgative gas flow.

### The Catalyst Dosing Measurement System

**Figure 7:**
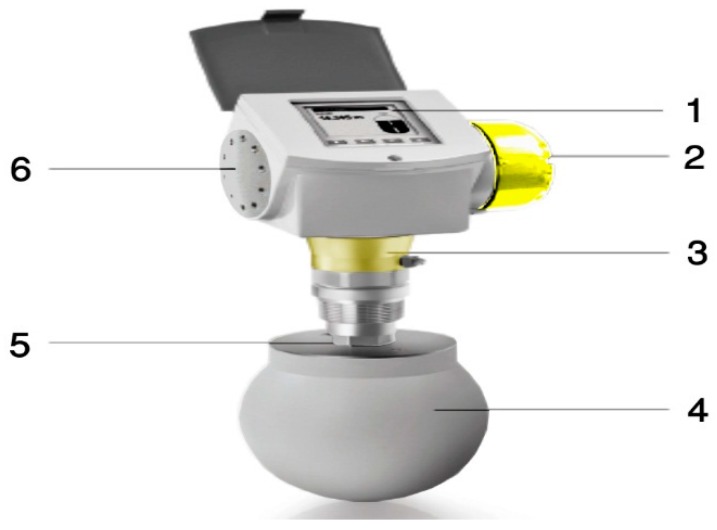
The catalyst dosing measurement device. (1) An elective touchscreen with a dose-controlling optional button; (2) A dual-wire reading meter; (3) A changeable and a rotatable converter consisting of a rapid connector technique; (4) Horn antennas (made of stainless steel); (5) A flange plate protector (needed for aggregating products) with extension services; (6) A single converter for many applications.

In the actual world, engineers find the measurement of the catalyst dosing in high pressure and heated polymerization reactor systems very difficult. In this report, we have reported the first device that was designed in the Department of Chemical Engineering, University of Malaya (UM). KROHNE Messtechnik GmbH, Germany, manufactured the specialized solid powder measurement device according to the request of UM. All the device features and components are presented in Table 4 and Figure 7. The device has been designed according to the FMCW (Frequency Modulated Continuous Wave), a radar level meter for measuring level, distance, volume and mass for several powder sizes, granules and all other solids. This form of measurement is more stable as compared to the pulse radar and is also better suited for dusty procedures. This device operates at high and low temperature values when the chemical process-connecting temperature values have been fulfilled.

## 4. Results and Discussion

Using the improved multiphasic phase and conventional mathematical model, the phenomena of gas-solid reaction with dynamic fluidization behavior modeling and simulation investigations of the propylene polymerization in the pilot scale fluidized bed reactor was conducted to prove the effects on the dynamic response and phase shift of the process of various hydrodynamic sub-models, model assumptions, and mixing conditions. To calculate the effect of key parameters like *U*_0_, catalyst dosing rate, monomer feed concentrations on the polypropylene production rate and fluidized bed dynamic situation during real reaction conditions, comparative and comprehensive simulations were done. Table 5 shows the operating conditions where simulations were carried out.

Given the advantages that the improved multiphasic model has over the prior ones, one can expect the improved model to give a result that is more realistic when compared to the conventional mathematical model. Moreover, it is worth noting that experimental validation of this type of model has been done for the first time. The results obtained exhibited the fact that this system’s improved multiphasic model agrees well with the experimental data.

### 4.1. Hydrodynamic Model in the Absence of Polymerization

**Figure 8:**
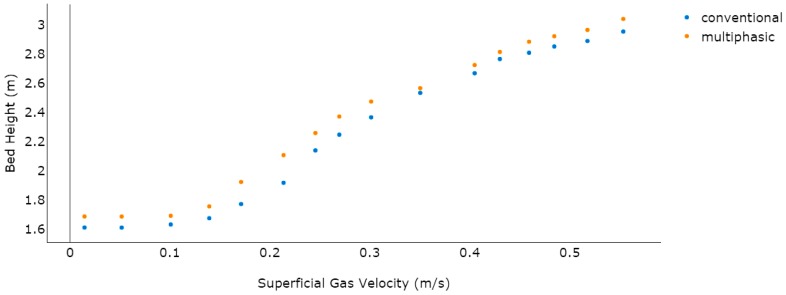
Dynamic bed behavior analysis between multiphasic and conventional model (without reaction).

In the process of devising an FBR, the pressure fluctuations are considered a critical parameter. They help determine the bubble dynamics in the system and quantify the intensity of the fluidization regime, even at oscillated velocity levels, by adjusting the bed height. At the pre-polymerization phase, bed height and pressure drop are vital parameters to examine the overall fluidization structure. To validate the proposed model, a CFD simulation between literature data and the developed model of the bed height *versus* superficial gas velocity are compared thoroughly. In this model, the top of the bed was set as a constant pressure outlet and the uniform inlet velocity was designed keeping in mind the inlet boundary conditions. The pilot-scale reactor has a cylindrical geometry containing the operational superficial gas with the velocity ranging from 0.2 to 0.6 m/s. We did not take into account the effects of front and back walls in this model. A no-slip wall boundary condition was used for the gas phase and a free-slip wall boundary condition was used for the solid phase. We assume that the bed is in the initial well-mixed condition and all velocities were set to zero at *t* = 0. The value of the void fraction was 0.53 and the static bed height was 1.5 m. The outlet pressure boundary condition was set at 25 bar. A detailed list of boundary conditions is provided in Table 5 and a dynamic correlation among these conditions is presented in Table 2. It was evident that a surge in the superficial gas velocity resulted in an increase in the bed height (see Figure 8).

Both the models proved to be in a good agreement with regards to bed expansion and the initial bed was predicted at 1.5 m in both. It was also noted that on increasing the superficial gas velocity, the maximum bed expansion for the available literature model reached 2.9104 m, while the multiphasic model’s highest bed expansion was found to be 3.1203 m, which proves the good agreement between the two models.

### 4.2. Bubble Emulsion Phase Distribution and Model Verification

Optimum propylene polymerization during the previous work (Khan, *et al.* 2016) [43] was discovered to have reached levels of around 6% per pass during the initial fluidization stage (Table 5 lists the simulated profiles). However, the very vital dynamic effects on the reaction rate were not considered in that work. By taking into consideration the dynamic parameters that are deemed as very significant process parameters for industrial-scale and commercial-grade propylene polymerization, this study has covered up that gap. As the reaction and fluidization proceed, the Ziegler-Natta catalyst, the catalyst feed rate, the superficial fluid velocity, and the monomer concentration in the reactor would change the fluidization dynamics. How these parameters are distributed in the reactor should therefore be investigated.

**Figure 9:**
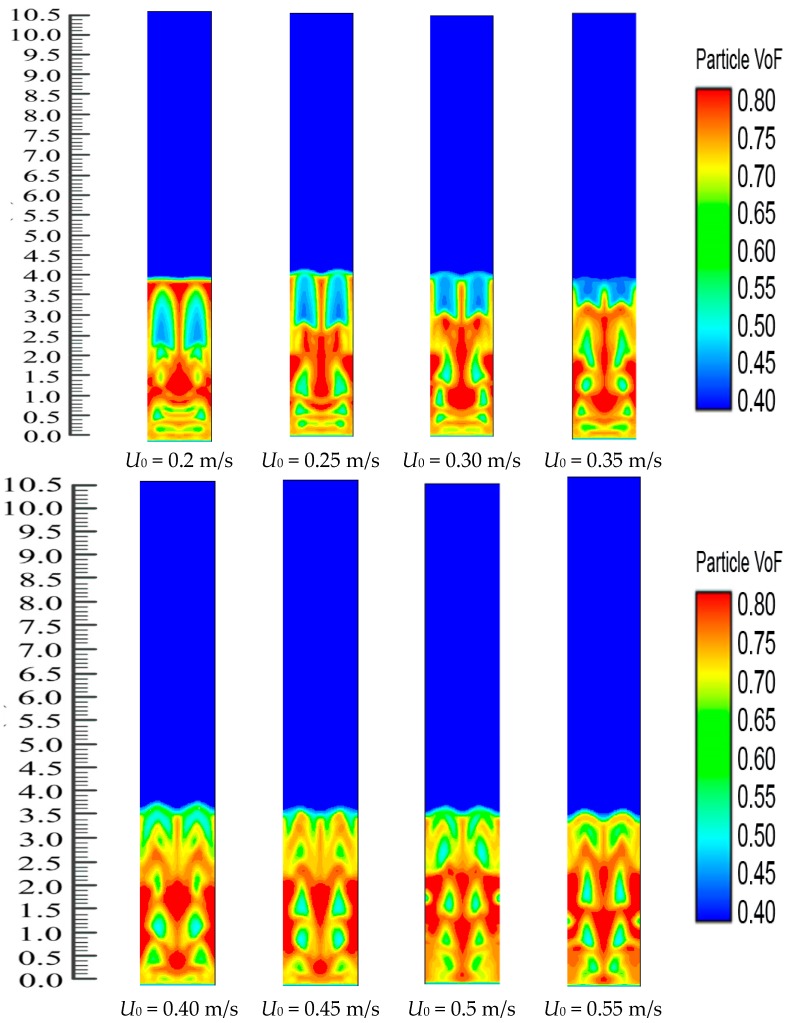
Dynamic effect of superficial gas velocity on phase (bubble and emulsion) formation before catalyst injection.

Polypropylene concentration distribution and bubble and emulsion phase formation in FBR at *U*_0_ = 0.2, 0.25, 0.3, 0.35, 0.4, 0.45, 0.50 and 0.55 m/s can be seen in Figure 9. Because the inlet reactants have the highest concentration, the heat supplied from the system heating source heats up the particles when the mixed gases come in contact with the bed particles. The figures clearly show that the gas-solid distribution exhibits significant dynamic changes in the reactor. These simulation-derived results can also give clear information on the conception of bubble and emulsion phase formation. Figure 9 clearly demonstrates that the change distribution and the bubble size are greatly altered with a variation in the U_0_ value. The bubbles present at *U*_0_ = 0.2 m/s are lesser than those in other situations. Herein, the bubbles form and move upwards in the reactor system but there was no bubble breakage. This ensured that there were more options present for the solid and the gas phase to come into closer contact. On the other hand, by the continued increments of the superficial fluid flow rate, the phenomenon of the bubble collapse can be clearly noted, which leads to a lesser chance for the close contact of the solid and gas phase. Theoretically, this phenomenon has been previously supported [47]. In this study, we have observed that the fluidized bed dynamics show a similar attitude when it reaches the *U*_0_ value of 0.4 m/s and continue until the value of U_0_ reaches 0.55 m/s. Figure 9 also shows the solid (bed particle) volume fraction development in the reactor where the average value is observed at 0.65 m/s. However, it is very important to determine if the *U*_0_ value has any impact on the propylene production rate or not. These issues have been highlighted in the subsequent section.

**Figure 10:**
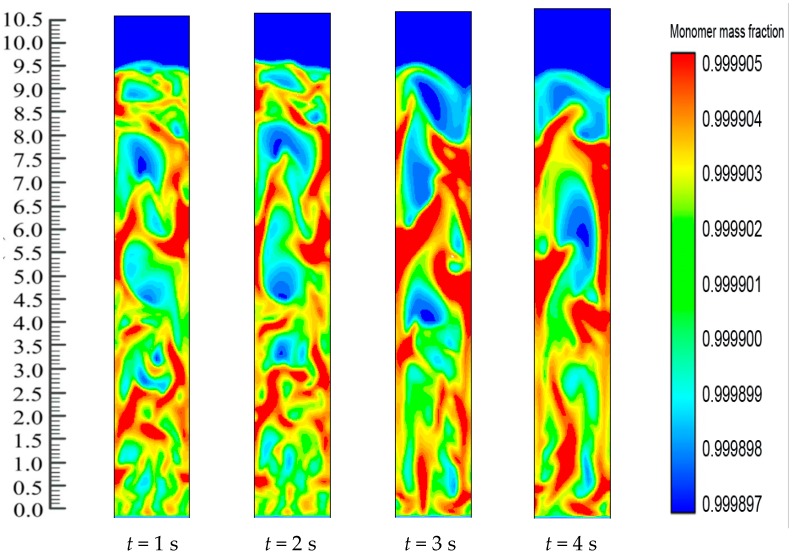

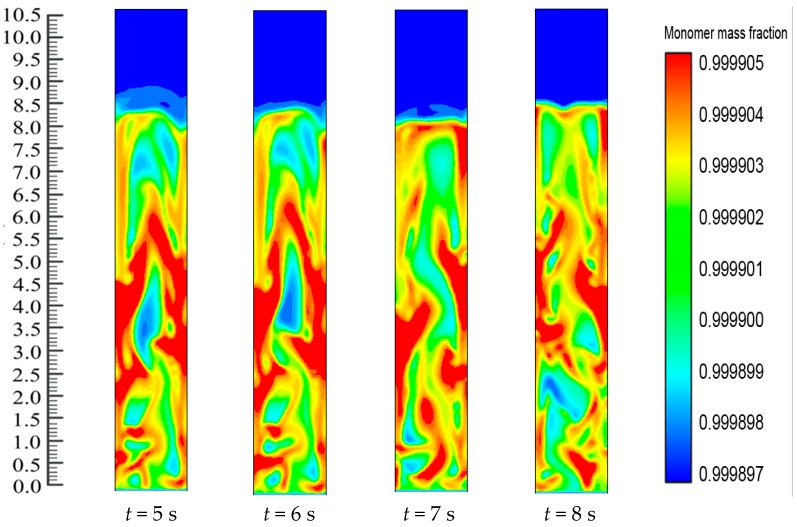
Mass fraction of monomer (propylene) during reaction at *U*_0_ = 0.2 m/s.

The fluidized bed dynamics after the catalyst’s injection in the system are depicted in Figure 10. Catalyst dosing immediately starts the exothermic reaction and releases energy from the reaction. The heat transfer from the particles then heats up the gases surrounding the bed particles and also results in differences in the production rates of the polymer. Thus, throughout the reaction system, there is a change in the mass fraction of the polymerized particle. Figure 10 shows the upward movement towards the bed of the polymer particle with the greater mass fraction. The figures also show the dynamic distribution in the FBR of the PP content profiles. This is due to the close positive relations among the reaction parameters. This also proves that dynamic catalyst activity determines the change in the gas/particle mass fraction in polymerization.

Figure 10 shows how, at the initial stage, the entire FBR has an identical propylene mass fraction distribution. The consumption of propylene and hydrogen and the generation of PP take place as the polymerization reactions go on. The distribution of the propylene mass fraction shifts until the flows of emulsion and bubble phases and the polymer distribution reach a stationary state (*i.e.*, 8 s). Along with the reactor height, the propylene mass fraction increases. This could be due to the fact that the hydrogen feed is limited and is consumed quickly to a relatively low level. Moreover, hydrogen has a significant impact on the reaction and deactivation rates. Under industrial conditions, a chain transfer with hydrogen is typically used to control the polypropylenes’ molecular weight, as this method is considered the most efficient. The ratio between the overall propagation rate and the total chain transfer rate determines the molecular weight of a polymer sample. However, this weight is not influenced by the polymerization activity. During this period, a relatively small amount of low molecular weight polymers are produced through the supplement of a large amount of hydrogen to the system. At higher temperatures, degradation reactions of cocatalyst compounds may generate chain termination agents.

**Figure 11:**
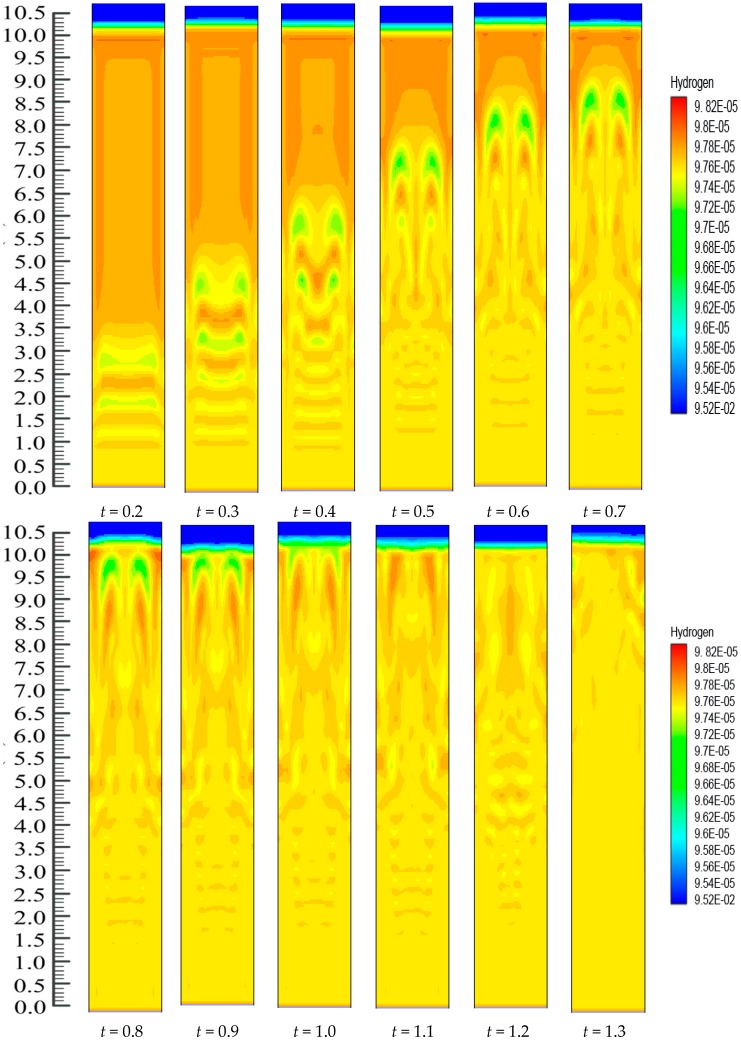
The dispersal transformation of hydrogen mass fraction due to alteration of time in the FBR at *U*_0_ = 0.2 m/s.

On the other hand, there is continuous polymerization to form PP. Moreover, since the FBR bottom has the highest catalyst concentration, the propylene mass fraction obtains a minimum at the FBR bottom, and shows a slight increase due to the decreases of the hydrogen mass fraction. The dynamic mass fraction for the hydrogen gas is illustrated in Figure 11. The hydrogen mass fraction goes on changing from the reactor bottom and it moves upwards in the system continuously. However, eventually, the mass fraction stabilizes at *t* = 1.2 s. However, it is worth mentioning that Figure 10 and Figure 11 illustrate the dynamic distributions of monomer (propylene) and hydrogen mass fractions, correspondingly. Additionally, one can also clearly compare the dominance of the propylene presence against hydrogen in the system from these snapshots, as it is very important in real reaction conditions to get a clear idea of this phenomenon. In literature it has been mentioned that the sum of the mass fraction values of propylene and hydrogen is near 1, which is in close agreement to our finding [48,49,50].

#### Model Validation Based on the Effect of Superficial Gas Velocity

**Figure 12:**
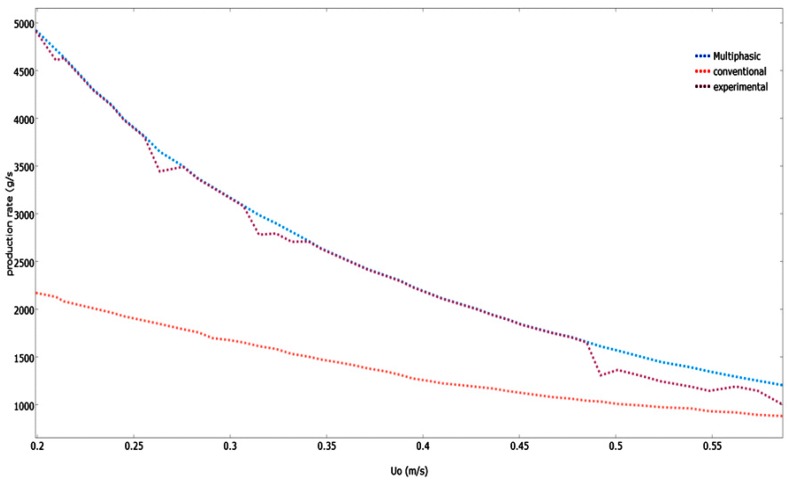
Effect of superficial gas velocity on the production rate (at optimum catalyst dosing 0.2 g/s).

The superficial gas velocity is an important process parameter because of its direct relationship to the propylene production rate, monomer residence time in the system, fluidization conditions, and particle mixing. It is therefore vital to study what effects it has on these process conditions. Figure 12 illustrates the various models that have predicted the impact of superficial gas velocity on the polymerization rate in the system.

As *U*_0_ increases, the polypropylene production rate drops. The monomer mean residence time decreases when there is an increase in the *U*_0_, which causes the monomer conversion and polymer production rate to decrease. Because the dominant emulsion phase is operating at conditions greater than the minimum fluidization velocity of gas, the production rate projected by the developed multiphasic model is greater than the conventional mathematical model. This leads to the emulsion phase having a lower void fraction and higher production rate. Compared to the predicted values of this multiphasic model, the experimental values were a little bit higher. Similar trends of production rates were also revealed. However, due to the fact that the emulsion phase starts at conditions beyond the minimum fluidization velocity of fluid, the polymerization rate projected by the conventional model is lower. As a result, the emulsion phase has a greater void fraction and lower reaction rate.

**Figure 13:**
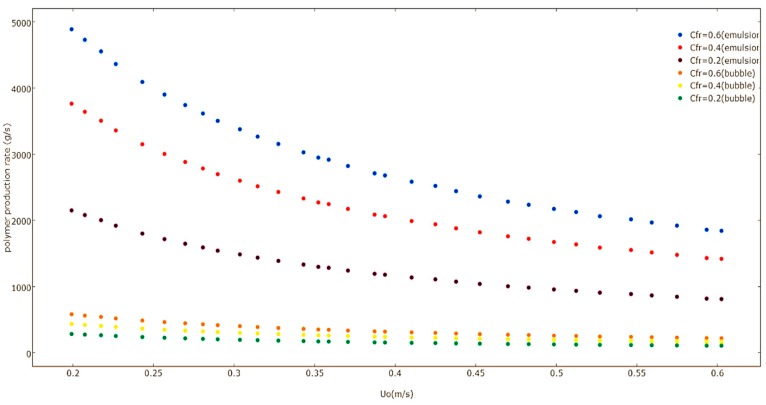
Effect of superficial gas velocity on the production rate of emulsion phases at various catalyst feed rates predicted by the multiphasic model.

Figure 13 illustrates the effect that *U*_0_ has on the polymerization rate of emulsion and bubble phases at varying Ziegler-Natta feed rates calculated by the multiphase model. The polymer production rate in the emulsion and bubble phases decreases when there is an increase in the value of *U*_0_, because the monomer mean residence time is decreased. This results in a lower polymer production rate.

**Figure 14:**
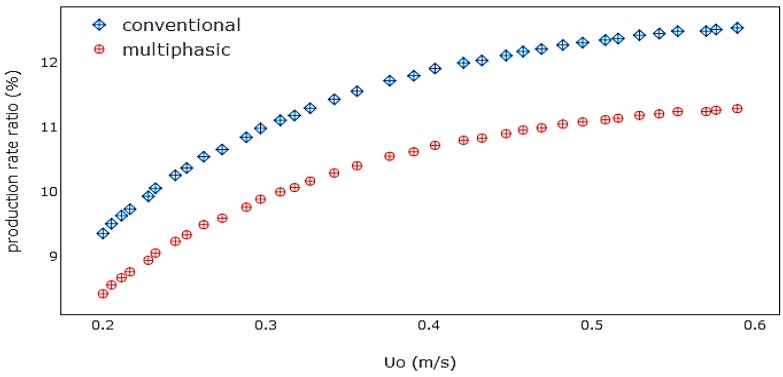
The ratio of polymer production in the bubble phase to the total production rate.

The multiphasic model was also used to predict the effect of superficial gas velocity on the polymer production rate by considering the proportion of the bubble phase polymerization rate to the overall polymerization rate, which is shown in Figure 14. The figure reveals how the increase of the superficial gas velocity results in an increase in the proportion of polymerization in the bubble phase over the total polymerization rate. When the superficial gas velocity is increased, more fresh reactant and solids enter the bubbles. This leads to a rise in the bubble impact on the polymerization rate. The bubble influence on the overall polymerization is approximately 9%–11%. This is already a noteworthy amount and it should be taken under consideration for a more consistent model projection. This model has underestimated the polymerization rate in the bubble, because, based on the bed hydrodynamics, it can clearly be observed that most of the reaction zone is occupied with a well-mixed emulsion phase. It is logical to assume that increased gas-solid contact results in the presence of larger amounts of catalyst. More space of contact between mixed active gases (propylene, hydrogen, and nitrogen) and catalyst can lead to an improved production rate.

To reduce the risks of agglomeration, high gas velocities are needed. However, the monomer conversion per pass through the reactor bed is reduced by high gas velocities and can result in greater elutriation of small particles from the bed.

### 4.3. Effect of Catalyst Feed Rate

Another key process parameter in the controlling of polypropylene FBCRs is the catalyst feed rate. Simplified hydrodynamic models do not take into account the presence of catalyst in the bubbles and consider that polymer production only takes place in the emulsion. However, the use of the multiphasic model made it possible to see that the emulsion phase contained about 91.7% of the catalyst while the bubbles had about 8.3% of the catalyst that was continuously charged into the reactor. The part of the reaction that takes place in the bubbles is therefore significant and must be considered.

When the fluidization is at a stable state, the polymerization reactions are at a steady state as well. In this case, the coupled model of the reacting flow can be verified using the product concentrations that are found in the FBR.

**Figure 15:**
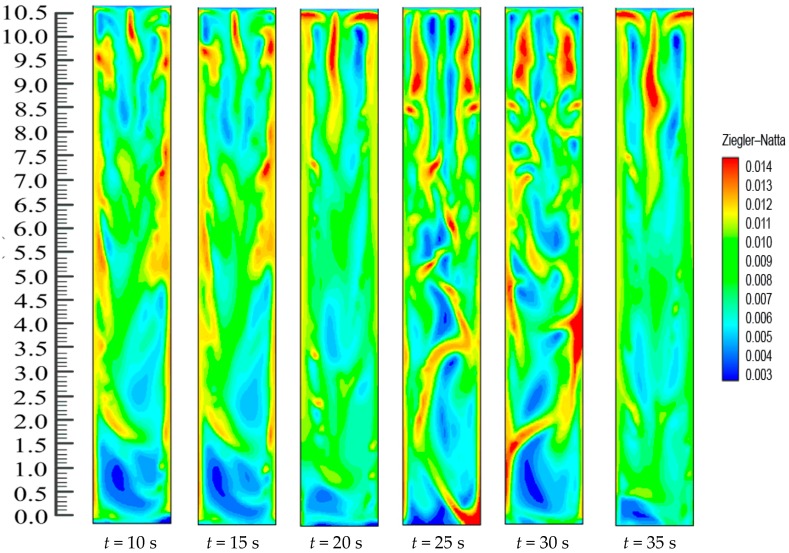
Snapshot of the solid volume fraction of catalyst particles with different time intervals in the FBR.

Figure 15 shows the catalytic dynamics. In the starting stage, catalyst particles that possess high active sites are produced at the bed bottom. Afterwards, particles with varying amounts are evenly mixed with gas. Solid also flows. In this situation, more polymer chain formation takes place by coordinating the monomer to the remaining active site of the Ziegler-Natta catalyst subsequently to insertion. Termination happens within a –β-hydride elimination process; consequently, the highest number of the chains is comprised of a terminal double bond. In this case, the sum of CH=CH_2_ groups is equivalent to the amount of methyl groups, which indicates that the chain transfer is progressing by β-hydride elimination. The activity of the catalyst particles in the FBR has been assumed to be taking place at a stable rate, but a snapshot of the catalyst dosing dynamics reveals that the activity is actually changing at a relatively slow pace because of the decentralized catalyst particles and unstable motion of the bubble. When hydrogen is consumed and its concentration along the bed height decreases, the rate of polymerization throughout the bed becomes high and results in a slightly higher catalyst presence on the upper part of the bed.

One of the great aspects of this model is that the selectivity of ethylene and propylene, and even the other species of conversion catalytic reactions, is almost identical in the FBR during the propylene polymerization process, given the real reaction conditions of higher active catalyst. Despite being deduced at a pilot scale, this CFD-kinetic model can still be utilized for industrial-scale reactors because the reactor type’s influence on the bulk reaction mechanisms can be neglected. The simulated data can be validated using the experimental data obtained from a practical FBR.

**Figure 16:**
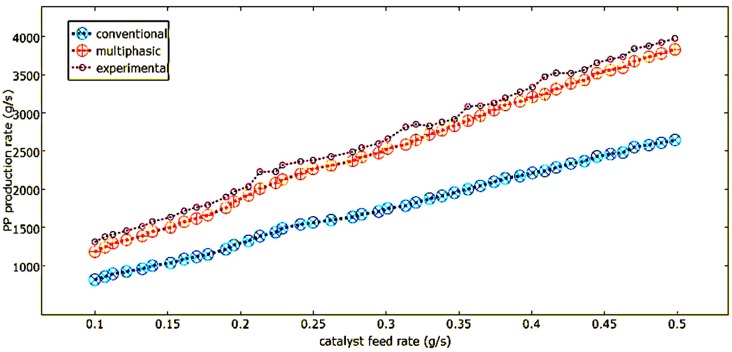
Effect of catalyst feed rate on polypropylene production comparison and validation.

In Figure 16, the effect that the catalyst feed rate has on the polymerization rate that the two models predicted, and the production dispersal rate in the phases that was calculated by the multiphasic model, can be observed. One can clearly observe how the polymer production rate and the catalyst feed rate are directly proportional. The polymer production rate increases when there is an increase in the catalyst feed rate because of the increase in the available active sites.

The improved multiphasic model predicts a polymer production rate that is lower than the conventional models in the bubble phase compared to the emulsion phase. This is because the improved dual-phase model takes into account the excess gas in the emulsion phase. This excess gas increases the void fraction and results in a decreased polymer production rate compared to the conventional models, which assume an emulsion phase that takes place at minimum fluidization. It can be seen in the improved dual-phase model that there is a higher rate of changes of production in the emulsion phase than that of the bubble phase. This is primarily due to the fact that there is more catalyst in the emulsion phase than in the bubble phase.

### 4.4. Effect of the Feed Composition

**Figure 17:**
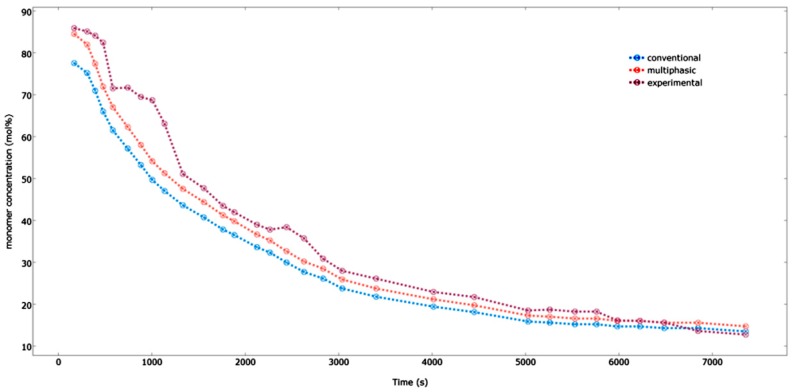
Effect of monomer composition (mol %) on polypropylene production.

Figure 17 demonstrates the comparison between the multiphasic and the conventional model results for the pilot plant data with respect to the propylene concentration within the reactor. As seen in the Figure 17, the predicted data for the multiphasic model agrees well with our experimental results, especially in the case of the long gap of the time points. The multiphasic model takes into account the solid particles present in the bubbles and the fact that the emulsion phase is at a condition which is beyond the minimal fluidization; hence, it provides more accurate and realistic results. On the other hand, the multiphasic model under-predicts the experimental results for shorter time durations. This is due to the fact that there is a very high heat and mass transfer rate between the bubble and emulsion phases in the beginning of the process, where the difference in the concentrations between both the phases is maximal. This situation, present in the initial fluidization stage, changes the reactor approach hydrodynamics to a well-mixed condition [21]. However, this type of mechanism becomes unrealistic further in the process as more and more bubbles are formed and the heat and mass transfer rate is decreased. The maximal variation that is seen between our experimental results and the predicted values for the multiphasic and the conventional model is approximately 3.0 and 4.5 mol %, respectively. This difference results because of the influence of the inert gases on the fluidized bed reactor’s hydrodynamic behavior.

## 5. Conclusions

A coupled CFD-dynamic mathematical model that assimilates the sub-models which describe the polypropylene production resulting from phase transition and gas–solid flow behavior in a gas-phase fluidized bed reactor was formulated. The dynamic bubble and emulsion phase concepts of fluidization served as bases for the hydrodynamics of the fluidized bed reactor of polypropylene production. This model was able to successfully capture the important flow features in a pilot-scale catalytic FBR. These features include the superficial fluid velocity, monomer-hydrogen concentration, catalyst feed rate, and the product concentrations inside the reactor at reacting-flow conditions. Moreover, analyses of the polymerization rate in individual phases, the monomer concentration in individual phase distributions in the reactor, and the ratio of polymer production in the bubble phase to the total production rate were done. An analysis of the effects of the main operation parameters on the reacting flow field was also done. A summary of the findings can be seen below.

(1) With the use of the multiphasic model, an investigation of the evolution of hydrodynamic phenomena in the FBR in typical fluidization states with different gas velocities was done. The developed model was also able to capture the gas-solid flow pattern, especially the solid flow pattern, something that was unobtainable using only the Eulerian–Eulerian method. This particular particle flow pattern promotes exceptional particle mixing, catalyst activation efficiency, and heat transfer, all of which are essential to the FBCR since the catalytic propylene polymerization process is an exothermic one.
(2) Under reaction-flow conditions, the simulation by the multiphasic model was conducted. Moreover, the effects of catalyst dosing, product mass fractions in the FBR at different regions, and the PP content bubble and emulsion phases were obtained. The results showed that the parameter distributions at different regions have significant differences for the polymerization process. The dynamic particle density distribution in the FBR is determined after injecting the catalyst and at various times. Because there is excellent contact within the catalyst and the solid and gas capability of the FBR, there is a uniform product fraction distribution in FBR.
(3) The conventional model was discovered to have predicted a lower emulsion phase production rate and propylene concentration under typical operating conditions. On the other hand, the improved multiphasic model agreed better with the experimental values. Compared to the conventional model, the improved multiphasic model also predicted a narrower and safer operation window at the same operating conditions. The improved multiphasic model showed that if one considers the practical range of the superficial gas velocity from 0.2 to 0.55 m/s and the catalyst feed rate from 0.2 to 0.6 g/s, the ratio of polymer production in the bubble phase to the total production rate will be calculated at around 9.4%–10.89%. This amount is significant and should be considered in the model. Moreover, it was revealed that the hydrodynamics and the reaction rate are strongly affected by the superficial gas velocity and catalyst feed rate. As a result, there is greater variation in the total production rate ratio. The improved multiphase model reveals that, at the beginning of polymerization, there is dynamic behavior that is close to the experimental results, but those figures also start to differ as the time increases.

In summary, this work has shown that a multiphasic polypropylene production model can be a useful guide in integrating process engineering efforts with reactor design efforts in the field of chemical engineering.

## Figures and Tables

**Table 1:** Kinetic mechanism of gas-phase catalytic propylene polymerisation

Reaction	Description	Rate coefficient	Unit	Value
$N\left(0,J\right)+M_{i}\overset{k_{i}(j)}{\to }N\left(1,j\right)$	initiation of polymerization	$k_{i}(j)$	m^3^·kmol^−1^·s^−1^	54.9
$N\left(r,J\right)+M_{i}\overset{k_{p}(j)}{\to }N\left(r+1,j\right)$	propagation	$k_{p}(j)$	m^3^·kmol^−1^·s^−1^	208.6
$N\left(r,J\right)+M_{i}\overset{k_{\text{fm}}(j)}{\to }N\left(1,j\right)+Q(r,j)$	chain transfer to monomer	$k_{\text{fm}}(j)$	m^3^·kmol^−1^·s^−1^	0.253
$N_{H}\left(0,J\right)+M_{i}\overset{k_{h}(j)}{\to }N\left(1,j\right)$	transfer to hydrogen	$k_{h}(j)$	m^3^·kmol^−1^·s^−1^	0.1
$N_{H}\left(r,j\right)+H_{2}\overset{k_{\text{fh}}(j)}{\to }N_{H}\left(0,j\right)+Q\left(r,j\right)$	transfer to hydrogen (cocatalyst)	$k_{\text{fh}}(j)$	m^3^·kmol^−1^·s^−1^	7.54
$N\left(r,J\right)+M_{\text{cat}}\overset{k_{\text{fcat}}(j)}{\to }N\left(1,j\right)+Q(r,j)$	transfer to catalyst	$k_{\text{fcat}}(j)$	m^3^·kmol^−1^·s^−1^	0.12

**Table 2:** Dynamic correlations and formulas applied for the multiphasic model [15,42,43].

Parameter	Formula
Bubble velocity	$v_{b}=v_{o}-v_{e}+v_{\mathrm{br}}$
Bubble rise velocity	$v_{\mathrm{br}}=0.7119{\left(gd_{b}\right)}^{1/2}$
Emulsion velocity	$v_{e}=\frac{v_{0}-\partial v_{b}}{1-\partial }$
Bubble diameter	$d_{b}=d_{\mathrm{br}}{\left[1+27(v_{0}-v_{e})\right]}^{1/3}(1+6.84H)$ $d_{\mathrm{br}}=0.0085$ (Geldard B category)
Bubble phase fraction	$\partial =0.534(1-e^{-\frac{v_{0}-v_{\mathrm{mf}}}{0.413}})$
Emulsion phase porosity	$\chi_{e}=\chi_{\mathrm{mf}}+0.2-0.059e^{-\frac{v_{0}-v_{\mathrm{mf}}}{0.429}}$
Bubble phase porosity	$\chi_{b}=1-0.146e^{-\frac{v_{0}-v_{\mathrm{mf}}}{0.439}}$
Volume of polymer phase in the emulsion phase	$\zeta_{\mathrm{pe}}=AH\left(1-\chi_{e}\right)(1-\partial )$
Volume of polymer phase in the bubble phase	$\zeta_{\mathrm{pb}}=AH\left(1-\chi_{b}\right)\partial$
Volume of the emulsion phase	$\zeta_{e}=AH\left(1-\chi_{b}\right)$
Volume of the bubble phase	$\zeta_{b}=A\partial H$
Minimum fluidization velocity	$\beta e_{\mathrm{mf}}={\left[{\left(29.5\right)}^{2}+0.357\text{Ar}\right]}^{1/2}-29.5$
Mass transfer coefficient	$K_{\text{sg}}=4.5(\frac{\beta e_{\text{mf}}}{d_{\text{pr}}})+5.85\left(\frac{PPC\cdot g^{1/4}}{d_{\text{pr}}{}^{5/4}}\right)$ $K_{\text{gs}}=6.77\left(\frac{D_{g}0.45v_{b}}{d_{\text{pr}}{}^{3}}\right)$
Momentum exchange coefficient	$K_{\text{mn}}=150\frac{\alpha_{s}^{2}v_{g}}{\alpha_{g}d_{\mathrm{pr}}}+1.75\frac{\alpha_{s}\rho_{g}}{d_{\mathrm{pr}}}\left|v_{b}-v_{e}\right|$

**Table 3:** Transport equations for dynamic multiphasic fluidized bed reaction system.

No.	Type of equations	Equations
1	General transport equation	$\frac{d\left(\alpha \rho \phi \right)}{dt}+\nabla .\left(\alpha \rho \vec{\upsilon }\phi \right)=\nabla .\tau^{\prime \prime }+S\phi$
2	The volume fraction dual-phase density	$\rho =\alpha_{2}\rho_{2}+\left(1-\alpha_{2}\right)\rho_{1}$
3	Momentum Equation	$\frac{d}{dt}\left(\rho \vec{\upsilon }\right)+\nabla .\left(\rho \vec{\upsilon }\vec{\upsilon }\right)=\;$−$\nabla p+\nabla .\mu [(\nabla .\vec{\upsilon }+{\vec{\upsilon }}^{T})+\rho \vec{g}+\vec{F}$
4.	The energy equation shared between the phases	$\frac{d}{dt}\left(\rho E\right)+\nabla .\left(\vec{\upsilon }(\rho E+p\right)=\nabla .k_{eff}\nabla T)+S_{h}$
5.	Inter-phase species transport equations	$\frac{d}{dt}\left(\rho Y_{i}\right)+\nabla .(\rho \vec{\upsilon }Y_{i})=$−$\nabla .{\vec{J}}_{i}+R_{i}+S_{i}$
6.	Mass transfer in bubble phase	Sb=Rbi(Mib±Mibp)
7.	Mass transfer in emulsion phase	Seu=Rei(Mie±Miep)
8.	The net velocity of the reactants	${\vec{u}}_{net}=\frac{\sum r\gamma_{j}^{r}M_{j}^{r}{\vec{u}}_{r,j}}{\sum r\gamma_{j}^{r}M_{j}^{r}}$
9.	Momentum transfer for the bubble phase	SbuU→=ℜi,p(MibU→net−MibpU→i)
10	Momentum transfer for the emulsion phase	SeuU→=ℜi,p(MieuU→−MieuU→i)

**Table 4:** Features of the catalyst dosing system.

Issues	Condition
accuracy	standard accuracy, ±10 n.gm (nano gram)/±0.4%
Inserted antenna/sensors	The shape prevents unexpected product build-up in complex dusty applications
Stability in extreme reaction conditions	Sensors can sustain at 200 °C(392 °F) temperature and 40 bar/580 psig pressure
Measuring range	Wide-ranged measurement capacity (up to 80 m/260 ft)
Data acquisition facility	Directly accessible graphic touchscreen/wizard (option 1) and optional second station (connected desktop computer) output
Prioritized particle	Ziegler-Natta catalyst

**Table 5:** System boundary and operating conditions used for simulation.

Factors	Value
Reactor volume	0.0215 m^3^
Initial bed height (m)	1.5
Initial void fraction	0.431
Gas density (kg/m^3^)	23.45
Catalyst diameter	3.0 × 10^−4^ m
Gas viscosity (Pa·s)	1.14 × 10^−4^
Mole fraction of hydrogen	2000 ppm
Cocatalyst concentration (mol/L)	0.01
Solid density (kg/m^3^)	1039 m^3^
Coefficient of restitution	0.8
Angle of internal fraction	30
Maximum solid packing volume fraction	0.75
Time step (s)	0.001
Activation energy, *E* (J·mol^−1^)	7.04 × 10^4^
Active site of catalyst (mol·m^−3^)	1.88 × 10^−4^
Feed monomer concentration (mol·m^−3^)	1.0
Hydrogen concentration (mol·m^−3^)	0.02
Inner diameter (Reaction zone)	0.1016 m
Cross sectional area	0.00785 m^2^
Height	1.5 m
Volume	0.011775 m^3^
Inner diameter (Disengagement zone)	0.25 m
Cross sectional area	0.0490625 m^2^
Height	0.25 m
Volume	0.0097 m^3^

## Nomenclature

${ℜ}_{r}$	instantaneous consumption rate of monomer (kmol/s)
$k_{f,r}$	Forward rate coefficient for reaction, *r*_th_
$\prod_{ct}$	molar concentrations of surface-adsorbed species on the catalyst active site
$\eta_{i,g,r}^{\prime }{}^{\prime }$	rate exponent for the *i*_th_ gas phase reactant
$\eta_{j,s,r}^{\prime }$	rate exponent for the *j*_th_ gas phase reactant
$C_{i}$	gas phase species
$S_{J}$	solid phase species
$R_{\;i,j(bu)}$	net molar rate of production in bubble phase
$R_{\;i,j(eu)}$	net molar rate of production in emulsion phase
$R_{\;i,j(ct)}$	net molar rate of production of catalyst active side for any phase
U_0_	Superficial gas velocity m/s
$B_{i,r}^{\prime }$	stoichiometric coefficient for each reactant species in bubble phase
$B_{i,r}^{\prime \prime }$	stoichiometric coefficient for produced PP in bubble phase
$E_{i,r}^{\prime \prime }$	stoichiometric coefficient for produced PP in emulsion phase
$E_{i,r}^{\prime }$	stoichiometric coefficient for each reactant species in emulsion phase
$C_{i,r}^{\prime }$	stoichiometric coefficient for each reactant species on catalyst active site
$C_{i,r}^{\prime \prime }$	stoichiometric coefficient for produced PP on catalyst
${\hat{ℜ}}_{i,p}$	monomer conversion rate
$N_{\text{as}}$	number of active site types
$M_{i}$	*i*-th component
$k_{p}$	propagation rate coefficient for a site of type *j* with terminal
$V_{R}$	reactor volume(m^3^)
$\varepsilon_{\min}$	void fraction of the bed at minimum fluidization
$U_{0}$	superficial gas velocity (m/s)
A	cross sectional area of the reactor(m^2^)
${\left[M_{i}\right]}_{in}$	concentration of component *i* in the inlet gaseous stream
$R_{v}$	volumetric polymer phase out flow rate from the reactor (m^3^/s)
$R_{i,p}$	instantaneous rate of reaction for monomer *i* (kmol/s)
$C_{\mathrm{pi}}$	specific heat capacity of component *i* (J/kg K)
$\rho_{\text{pp}}$	density of mixed gas density(kg/m^3^)
$C_{p,\mathrm{sol}}$	specific heat capacity of solid product (J/kg K)
$T_{\text{in}}$	temperature of the inlet gaseous stream (K)
$T_{r}$	Average temperature in the bed at (K) during $\varepsilon_{\min}$
$R_{p}$	production rate (kg/s)
$\rho_{\mathrm{eu}}$	Average density of emulsion phase
φ	phase variable
α	phase volume fraction
ρ	the phase density
$\vec{\upsilon }$	phase velocity
$\tau^{\prime \prime }$	the diffusion term
Sφ	the source term
*E*	Phase energy
$k_{\text{eff}}$	effective thermal conductivity
$S_{h}$	volumetric heat sources
$R_{i}$	net rate of production of species *i*
$S_{i}$	rate of *i*, species transport
*j*	*J^th^* item (either a reactant or a product).
$H_{\vec{b}e}$	bubble to emulsion heat transfer coefficient(W/m^3^ K)
$U_{b}$	bubble velocity (m/s)
$\rho_{\mathrm{pol}}$	polymer density(kg/m^3^)
$C_{p,\mathrm{sol}}$	specific heat capacity of solid product (J/kg K)
$\varepsilon_{\mathrm{bu}}$	void fraction of bubble for Geldart B particles
$\rho_{\mathrm{pol}}$	polymer density(kg/m^3^)
$C_{p,\mathrm{pol}}$	specific heat capacity of solid product (J/kg K)
$\Delta H_{R}$	heat of reaction (J/kg)
$\alpha_{\mathrm{bu}}$	volume fraction of bubbles in the bed
$\left[M_{i}\right]$	“I” monomer concentration (molMe/m^3^)
P_0_	active sites concentration (kmol/m^3^)
N(0, j)	uninitiated site of type *j* produced by formation reaction
N(r,j)	living polymer molecule of length r, growing at an active site of type *j*, with terminal monomer M
N_H(0,j)_	uninitiated site of type *j* produced by transfer to hydrogen reaction
*r*	number of units in polymer chain
*k*_fm__(j)_	transfer rate coefficient for a site of type *j* with terminal monomer M reacting with monomer M
*k*_fcat__(j)_	transfer rate coefficient for a site of type *j* with terminal monomer M reacting with catalyst
*k*_h__(j)_	rate coefficient for reinitiating of a site of type *j* by monomer M
*k*_i__(j)_	rate coefficient for initiation of a site of type *j* by monomer M
*k*_p__(j)_	propagation rate coefficient for a site of type *j* with terminal monomer M reacting with monomer M
PBM	Population Balance Model
PMLM	Polymeric Multilayer Model
Subscripts and superscripts	
*b*_u_	bubble phase
*e*_u_	emulsion phase
g	gas mixture property
*i*	component type number
in	inlet
*j*	active site type number
pol	polymer
ref	reference condition
